# Linking Iron Metabolism, Ferroptosis, and Cancer: New Targets and Prospects for Effective Anticancer Therapeutic Interventions

**DOI:** 10.3390/cancers18091436

**Published:** 2026-04-30

**Authors:** Malamati Kourti, George J. Kontoghiorghes

**Affiliations:** 1 Postgraduate Research Institute of Science, Technology, Environment and Medicine, 3021 Limassol, Cyprus; m.kourti@euc.ac.cy; 2 Department of Life Sciences, European University Cyprus, 2404 Nicosia, Cyprus

**Keywords:** cancer, iron, ferroptosis, iron chelation therapy, tumour microenvironment, personalised medicine, deferiprone

## Abstract

New anticancer therapeutic strategies, including targeting of iron dysregulation in affected cancer types and stages, are urgently needed to decrease the associated annual cancer death rate of about 10 million worldwide. Many tumours evade treatment and support metastatic potential by effluxing iron and upregulating antioxidant systems, leading to suppression of lipid peroxidation and ferroptotic cell death. Similarly, many tumours manipulate the tumour microenvironment (TME) by ensuring the continuous supply of iron. This involves phenotypic modulation of immune cells, including macrophages, neutrophils, regulatory T lymphocytes, and natural killer cells, as well as fibroblasts, contributing to immune evasion and tumour growth. In particular, tumour-associated macrophages (TAMs), which may account for about half of the tumour’s bulk, become progressively heavily loaded with iron and can be detected by magnetic resonance imaging (MRI) technologies. Clinically effective iron chelation therapy protocols in iron-overloaded conditions using the chelating drugs deferoxamine, deferasirox, and especially deferiprone can also potentially remove excess iron from TAMs and may decrease tumour malignancy. Deferiprone can also remove excess iron from iron-loaded renal cancer cells and potentially prevent metastasis in renal carcinoma. The anticancer potential of deferiprone has also been shown in other cancers, including iron removal in prostate cancer and through cancer stem cell inhibition in breast cancer. Many ongoing clinical trials using different drugs and experimental agents for inducing or modulating ferroptosis also support the translational potential of ferroptosis-based therapeutic strategies in selected categories of cancer patients. These advances highlight ferroptosis as a potential key metabolic vulnerability with relevance for treatment-resistant and metastatic tumours. Overall, iron chelation therapeutic approaches and ferroptosis-targeting may be considered for significant use as monotherapies or in combination with other anticancer drugs and could potentially improve therapeutic outcomes and limit disease progression and mortality in many cancers.

## Simple Summary

Cancer cells require large amounts of iron to grow and spread. Many cancers adapt by controlling iron levels through the modulation of immune cells and fibroblasts in their tumour microenvironment, which also allows them to survive treatment and metastasise. This review explores how iron metabolism and ferroptosis are closely linked to cancer progression, immune evasion, and resistance to therapy. It also discusses emerging treatment strategies in selected cancer types and stages that either cause the removal of excess iron from the tumour microenvironment using potentially efficient chelating protocols of deferiprone, deferoxamine, and deferasirox or by triggering ferroptosis to kill cancer cells. By targeting these vulnerabilities in selected cancers, new and combined therapies may potentially improve treatment effectiveness. Overall, this work highlights iron regulation and ferroptosis as promising targets for developing more precise and effective cancer treatments.

## 1. Introduction

Cancer is considered one of the leading groups of diseases affecting humanity, with a high rate of morbidity and mortality, as well as wide-ranging detrimental social implications. It is estimated that in recent years, the worldwide number of new cancer cases per year has been about 20 million and related deaths about 10 million. This number is likely to increase substantially in the next 25 years, particularly in developing countries, where cancer screening, diagnosis, and treatment are inferior to those in developed countries due to a lack of resources [1,2,3,4,5].

Despite many advances in anticancer research, further efforts are needed to substantially reduce cancer patients’ morbidity and mortality, including the development of new strategies related to its prevention, diagnosis, and treatment. With regard to cancer treatment, there are many complexities and limitations that make such efforts difficult. In particular, the therapeutic approach for each type and stage of cancer differs, with specific targeting and treatment requirements in each case.

There are many other major complications affecting therapeutic outcomes, which in many cases limit the efficacy and tolerance of anticancer treatments, such as drug resistance and toxicity. For example, it is estimated that about 90% of cancer patients experience a reduction in anticancer drug efficacy due to drug resistance. This drawback is observed in both older and newer categories of drugs, including immunotherapy agents [6,7,8,9,10]. Several mechanisms appear to be adopted by cancer cells in the development of drug resistance, such as increased drug efflux, enhanced drug metabolism, altered metabolism of nutrients and metal ions, genetic and immunological changes, and increased DNA repair capacity [6,7,8,9,10].

Anticancer drug toxicity is another major area under investigation, which greatly affects the morbidity and mortality of cancer patients. Different levels of toxicity are observed in most patients treated with anticancer drugs, ranging from mild to fatal side effects. In most cases, the toxicity arises mainly from the damaging effects of anticancer drugs on normal cells, affecting the physiological function of different organs [11,12,13,14].

The most advanced stage of malignancy in cancer patients is tumour metastasis, which develops through a multistep process that begins at the primary tumour site and accounts for more than 90% of cancer-related mortality. In tumour metastasis, cancer cells break away, travel through the bloodstream or lymphatic system, and migrate to selected distant organs where they form secondary tumours. The treatment of metastasis is very difficult and often impossible [14,15,16,17,18,19,20].

Thousands of potential methods and applications, including new targeted therapeutics, are being developed to decrease malignancy at different stages and in different types of cancer, with the main aim of increasing survival and improving the quality of life of cancer patients. One of the main targets is the modulation of iron toxicity, metabolism, and associated processes such as ferroptosis, which appear to play a pivotal role in selected types and stages of cancer, including initiation, proliferation, immune evasion, metastasis, and drug resistance [21,22,23,24,25,26,27,28].

Some of the major hallmarks associated with the involvement of iron in cancer include increased demand and rate of iron uptake, as well as changes in key protein turnover in cancer cells. Similarly, relevant changes in the tumour microenvironment (TME) are observed to ensure the continuous supply of iron to tumours, particularly in iron-dependent malignancies. These include phenotypic modulation of immune cells and fibroblasts, among others. In particular, the progressive modulation of iron metabolism, including increased iron loading of macrophages in the TME, contributes to tumour progression and highlights the participation of iron in enhancing malignancy.

In contrast, modulation of iron metabolism, ferroptosis, and oxidative stress are closely linked not only to therapy resistance but also to tumour progression and metastasis. In particular, iron-driven lipid peroxidation appears to promote cancer development and, when excessive, trigger ferroptotic cell death, creating a therapeutic vulnerability. However, many tumours evade treatment and support metastatic potential by upregulating antioxidant systems, which suppress lipid peroxidation and ferroptosis.

Targeting all the above and other related pathways, such as modulating iron metabolism or inducing ferroptosis in tumours, could reduce tumour survival and metastasis. Furthermore, combining iron metabolism modulators and/or ferroptosis inducers with existing therapies could provide an additional strategic approach against tumour progression.

**Figure 1:**
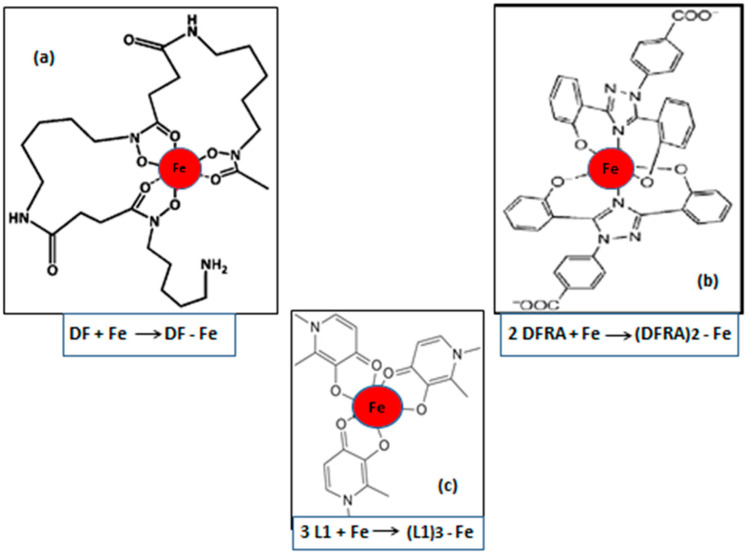
The structure of iron complexes following the reactions of the chelating drugs deferoxamine, deferiprone, and deferasirox with iron. At physiological pH, iron (Fe) reacts with deferoxamine (DF), forming a 1:1 molar ratio stoichiometry iron complex (**a**), with deferasirox (DFRA), a 2:1 molar ratio stoichiometry iron complex (**b**), and with deferiprone (L1), a 3:1 molar ratio stoichiometry iron complex (**c**). All chelating-drug iron complexes have an octahedral structure with iron (Fe) in the centre, depicted as a red sphere. Abbreviations: DF: Deferoxamine, DFRA: Deferasirox, L1: Deferiprone, Fe: Iron.

Clinical trials investigating iron metabolism modulators, including the iron-chelating drugs deferiprone (L1), deferoxamine (DF), and deferasirox (DFRA) (Figure 1), as well as ferroptosis-targeting agents, are already underway, highlighting their potential for future cancer treatment. Overall, iron metabolism modulators and ferroptosis-based therapies represent a novel and promising approach to improve cancer treatment outcomes in molecularly and metabolically defined subsets of cancer. Furthermore, the selection of appropriate targeting strategies, for example, prioritising specific therapies for each type and stage of cancer based on personalised medicine parameters, could lead to more effective and less toxic therapeutic outcomes.

In recent years, several comprehensive reviews have addressed ferroptosis as a regulated form of cell death in cancer, with emphasis on lipid peroxidation pathways, antioxidant systems such as the solute carrier family 7 member 11 (SLC7A11)/glutathione peroxidase 4 (GPX4) axis, and emerging ferroptosis-inducing agents. In parallel, other reviews have discussed cancer-associated iron dysregulation, focusing on altered iron uptake, storage, and systemic iron biomarkers. However, these topics are often examined in isolation, with limited integration of iron metabolism, ferroptosis regulation, and TME dynamics across different cancer types and stages. Moreover, comparatively less attention has been given to clinically actionable strategies that exploit iron dependence, particularly the modulation of iron pools within tumour cells and TME-associated immune cells.

The present review aims to bridge this gap by providing an integrated and clinically oriented synthesis of iron metabolism and ferroptosis in cancer progression, immune evasion, metastasis, and treatment resistance. In contrast to prior reviews, particular emphasis is placed on (i) the role of iron redistribution within the TME, especially tumour-associated macrophages (TAMs) and fibroblasts, (ii) the dual and context-dependent functions of iron-driven oxidative stress in promoting malignancy versus triggering ferroptotic cell death, and (iii) translational and ongoing clinical efforts targeting iron metabolism and ferroptosis, including iron-chelation strategies and combination approaches. By positioning ferroptosis within the broader framework of iron regulation and personalised therapeutic targeting, this review seeks to complement existing literature and highlight iron metabolism as a central, yet underexploited, vulnerability in selected cancer types and stages.

The main purpose of this review is to outline the diverse roles and mechanisms of iron metabolism and ferroptosis across different cancer types and stages of malignancy, with emphasis on recent advances that extend and update existing reviews. In particular, this review focuses on emerging clinical trial efforts, the crucial role of TME modulation, and evolving strategies aimed at controlling metastasis and treatment resistance. Furthermore, it discusses novel anticancer strategies targeting iron metabolism and ferroptosis, including the use of ferroptosis inducers and modulators of iron homeostasis, with special reference to the clinical effects of iron-chelating drugs in selected cancer types. The potential integration of iron-targeting approaches with existing anticancer therapies is also examined, within the context of personalised medicine and including previous clinical applications of iron-chelating drugs in iron overload and other related diseases [21,29,30,31].

## 2. Iron in Cancer: Roles and Hallmarks

Iron plays an essential role in many metabolic processes in cancer cells, where the requirements for iron are generally much higher than those observed in normal cells because of rapid growth and proliferation, as well as the modulation of other activities, including immune evasion, malignant transformation, metastasis, and drug resistance [21,32].

### 2.1. Iron Metabolism Changes in Cancer Cells and the Tumour Microenvironment

Reprogramming and modulation of normal iron metabolism are observed in different types of cancer cells, leading to an increased rate of iron uptake and utilisation, as well as controlled iron efflux, so that toxicity from intracellular iron accumulation, ferroptosis, or other forms of oxidative damage in cancer cells is prevented [21,27,28,32]. The impact of cancer on iron metabolism is characterised by many changes, including, for example, the observation that iron deficiency is manifested in many categories of cancer patients during cancer progression and metastasis [26,33].

Specific host cell populations and various mechanisms are adopted by cancer cells or introduced in the TME to facilitate and maintain a continuously increased supply of iron to tumours. In particular, major changes are observed in immune cells and cancer-associated fibroblasts (CAFs), both of which contribute to the modulation of iron metabolism in the TME and also to extracellular matrix remodelling. These modifications further facilitate the growth and proliferation of tumours and also help tumours achieve, among other effects, immune evasion, as well as other changes such as the development of metastasis and drug resistance [34,35].

Cancer-associated fibroblasts represent a high proportion of non-cancer cells in the TME. They appear to be derived from different categories of cells, including fibroblasts, epithelial cells, endothelial cells, cancer stem cells, and adipocytes, each of which has functional diversity and different pro-tumourigenic roles that contribute to extracellular matrix remodelling and tumour development and expansion. In particular, the activation of CAFs by iron-catalysed reactive oxygen species (ROS) also appears to be involved in the promotion of tumour metastasis [34,35,36].

In addition to CAFs, a variety of other cells, extracellular matrix proteins, and other chemical components and factors appear to contribute to tumour growth and development [36,37]. Hypoxia, for example, is a major characteristic of the TME, where iron plays a major role in promoting the proliferation of hypoxic cancer cells through different mechanisms. These mechanisms include the enhancement of iron utilisation and storage, as well as the controlled efflux of iron through ferroportin and exosomes, all of which further contribute to cancer cell proliferation, immune escape, metastasis, drug resistance, and ferroptosis resistance [21,38,39,40,41].

Another major characteristic of the TME is acidity, which appears to be caused by the pumping out of protons and lactic acid by cancer cells in tumours, while at the same time an alkaline environment is maintained intracellularly [37,42,43]. Under these conditions, the acidity in the TME (pH 5.5–7.0) increases iron solubility and enhances iron availability for tumour growth. It should be noted that at such low pH values, many other iron metabolic pathways are also affected. For example, iron is released from transferrin in the acidic (pH 5.5) environment of endosomes, following intracellular incorporation of the transferrin receptor–transferrin–iron complex [44]. Similar molecular mechanisms leading to an increased rate of iron release in acidic media, such as the TME, are observed not only from transferrin but also from other iron-containing proteins, such as lactoferrin, which is found in different bodily secretions and stored in the secondary granules of neutrophils, as well as ferritin and hemosiderin. In contrast, under alkaline conditions such as those predominating in tumours, the rate of low-molecular-weight labile iron catalytic forms decreases, and the rate of redox activity, including that associated with ferroptotic cell death, is likely to be inhibited or reduced.

### 2.2. Iron Metabolism Changes in Immune Cells of the Tumour Microenvironment

There are many links and influences between iron metabolism, immunity, and cancer progression, with immune cells playing an important role in various activities associated with the TME in addition to CAFs. In this context, major changes in iron metabolism, including those related to immune cells and overall immune function, are observed in the TME, which may lead to tumour progression, metastasis, and drug resistance [36]. Despite the general trend for increased requirements for iron supply in all categories of cancer cells, their dependence on iron and the changes in iron metabolism in each category are diverse, resembling those found in different categories of normal cells with variable iron needs, such as, for example, increases in iron uptake and utilisation in hemopoietic cells.

The presence and function of different immune cells and other non-cancer cell categories in the TME are critical parameters for the development and progression of many categories of tumours. In these cases, different immune cell types appear to infiltrate the tumours, including TAMs, neutrophils, B and T lymphocytes, and natural killer (NK) cells, the function of all of which is progressively modulated in the TME in order to support the growth and development of tumours. In particular, the presence of immune cells in the TME appears to be essential for tumour development, especially considering that it has been suggested that TAMs alone account for about half of the tumour’s bulk in most solid tumours and also many haematological malignancies [36,45,46,47,48].

Under normal physiological conditions, macrophages play an important role in the phagocytic system of many organs such as the spleen and liver, where, for example, effete red blood cells are phagocytosed, with haem being metabolised and iron stored and/or released into the circulation and then to the tissues via transferrin [49]. Similarly, damaged or dead cells, including those involved in ferroptosis, are also phagocytosed by macrophages. However, a different pathway is observed in anaemia of inflammation (also called anaemia of chronic disease), where iron is diverted and stored in macrophages of the reticuloendothelial system, thus reducing iron availability to the haemopoietic tissues and causing anaemia [50,51]. Similar pathways diverting iron and leading to anaemia are also observed during infections and cancer.

Macrophages are a heterogeneous population of highly plastic cells, with a spectrum of functional states that vary according to tissue distribution, functional state, response to the microenvironment, and other factors [50,52,53]. In general, macrophages can be functionally polarised into the classical M1-like state, which is pro-inflammatory, and the M2-like state, which is alternatively activated, with their functional phenotype modified by different factors, including their interaction with other immune cells, cancer cells, or pathogens. The role of M1 and M2 macrophages in iron metabolism is also different, with macrophage polarisation affecting iron homeostasis [54]. In this context, M1 macrophages are characterised by high ferritin and low ferroportin expression and increased production of reactive oxygen and nitrogen species. They are involved in marked iron sequestration, retention, and storage in the anaemia of chronic disease, as well as in bacteriostatic and bactericidal activity, immunostimulation, and tumour suppression. In contrast, M2 macrophages are characterised by low ferritin and high ferroportin expression and are involved in iron release, immunoregulatory function, tissue repair, matrix remodelling, and tumour promotion [50,52,53].

Macrophages also play an important role in linking inflammation and cancer. In solid tumours, TAMs are also heterogeneous, characterised by an alternative-like activation phenotype with great plasticity. In the TME, M1, and M2 macrophages also appear to function differently, with M1 macrophages progressively becoming iron-loaded, thus impeding their tumour-suppressive activity, while at the same time, M2 macrophages act as an iron reservoir for the continuous supply of iron to support tumour growth. In the latter case, ferroportin has been shown to play a critical role in the malignant potential of tumours due to increased iron availability in the TME and tumour cells [55].

Many other immune cells also appear to be involved in the development and growth of tumours by utilising different iron metabolic pathways. For example, tumour-associated neutrophils (TANs), which are characterised by phenotypic and functional plasticity similar to TAMs, appear to play an important role in the TME [56,57,58,59,60,61]. Under certain conditions, TANs appear to secrete transferrin, which is identified as a key regulator of metastatic tumour cell growth through iron accumulation [62]. Similarly, excess iron accumulation makes TANs susceptible to ferroptosis, which represents a further source of iron for tumour growth and immunosuppression [56]. In particular, TANs are known for their pro-tumour characteristics and seem to play a critical role in the progression and prognosis of triple-negative breast cancer (TNBC), where ferroptosis induction is accomplished via ferritin heavy chain (FTH) [63]. It is interesting that FTH supports the stability and function of regulatory T cells (Treg), which express iron regulatory genes, including those encoding FTH. It appears that FTH expression in Treg is essential for immune homeostasis [64]. However, in oral squamous cell carcinoma and other cancers, FTH is considered a tumour biomarker associated with cancer proliferation and migration [65]. In another study analysing solid tumour datasets of many cancers, ferritin light chain (FTL) and FTH levels appear to correlate with tumour infiltration by TAMs and Treg cells. This suggests that FTL, FTH, and iron play an important role in regulating tumour immunity in solid tumours [66].

Another category of immune cells that plays an important role in the anti-tumour response is NK cells, which are part of innate immunity and are engaged in crosstalk with other immune cells and CAFs present in the TME [67,68,69]. Under these conditions, CAFs appear to inhibit NK cell activity against tumour progression and immune escape by modulating iron metabolism and inducing ferroptosis in NK cells [70]. In contrast, iron depletion and FTH downregulation increase the susceptibility of both primary cancer cells and macrophages to NK cell recognition and activation [71].

The overall modulation of immune cell activity, iron metabolism, and ferroptosis in the TME appears to be directly related to the development and progression of tumours, as well as to many pro-tumour activities, including immune evasion, malignant transformation, metastasis, and drug resistance.

## 3. The Role of Iron in Ferroptosis and Oxidative Stress in Cancer

Ferroptosis is an iron-dependent cell death program that is morphologically, biochemically, and genetically distinct from apoptotic and other cell death processes [21,22,72]. It is mainly characterised by, among other features, an increase in intracellular labile iron as a result of ferritinophagy, sustained production of free radical reactions and lipid peroxides catalysed by labile iron, inhibition of GPX4 activity, and a decrease in glutathione (GSH) production [21,22,72].

Iron plays a central role in oxidative stress and lipid peroxidation, which are essential biochemical processes driving ferroptosis. In normal and cancer cells, dysregulated iron metabolism may result in an expanded labile iron pool (LIP), increasing the availability of redox-active iron capable of catalysing free radical reactions. Ferrous iron (Fe^2+^) participates in Fenton and Haber–Weiss reactions, generating highly reactive hydroxyl radicals (•OH) and other ROS that initiate and propagate oxidative damage to cellular macromolecules, particularly polyunsaturated fatty acids (PUFAs) within membrane phospholipids [22,73].

Free radical formation in lipids catalysed by iron leads to the accumulation of lipid hydroperoxides, which, when not adequately detoxified by antioxidant systems such as GSH and GPX4, compromise membrane integrity and result in cell death [74]. Cancer cells, especially those with high metabolic activity and increased PUFA content, are particularly vulnerable to iron-mediated lipid peroxidation [75,76]. However, many tumours develop adaptive mechanisms that enhance antioxidant defences or alter iron trafficking to avoid ferroptotic death [77].

**Figure 2:**
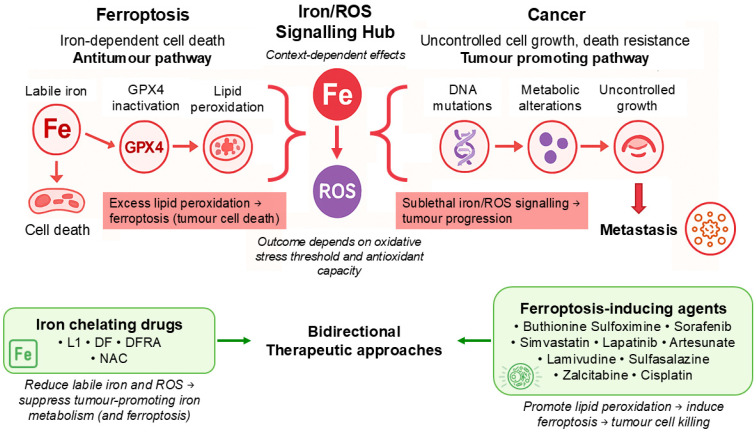
Context-dependent interplay between ferroptosis, iron metabolism, and cancer progression with associated therapeutic strategies. Iron metabolism and ROS act as a central hub linking ferroptosis and cancer progression in a context-dependent manner. Increased labile iron promotes ROS generation, which can drive lipid peroxidation. When lipid peroxidation exceeds the cell’s antioxidant capacity due to reduced GPX4 activity and/or glutathione depletion, it leads to ferroptosis, an iron-dependent form of cell death that contributes to tumour suppression. However, when iron-dependent oxidative stress is not sufficient to trigger ferroptosis, it can instead promote cancer development. In this setting, sublethal ROS levels contribute to DNA damage, metabolic reprogramming, and uncontrolled cell growth, ultimately supporting tumour progression and metastasis. These opposing outcomes reflect a balance between oxidative stress and antioxidant defence systems within the cell. Cancer cells often adapt by strengthening antioxidant pathways, allowing them to avoid ferroptosis while still benefiting from iron-driven processes that support growth and survival. Therapeutically, this pathway can be targeted in different ways, always based on diagnostic criteria. Ferroptosis-inducing agents (such as buthionine sulfoximine, sorafenib, sulfasalazine, and cisplatin) promote lipid peroxidation to trigger cancer cell death. In contrast, iron-chelating agents (including deferiprone, deferoxamine, and deferasirox) reduce iron availability and ROS production, thereby limiting tumour-promoting effects of iron metabolism, even though they also inhibit ferroptosis. Together, these approaches highlight the dual and context-dependent role of iron and ferroptosis in cancer, and the need to tailor therapeutic strategies accordingly based on each patient’s diagnostic markers. Abbreviations: DF: Deferoxamine, DFRA: Deferasirox, GPX4: Glutathione Peroxidase 4, L1: Deferiprone, NAC: N-acetylcysteine, ROS: Reactive Oxygen Species.

Oxidative stress induced by iron is not entirely detrimental but may also, under certain conditions, contribute to tumour initiation, genomic instability, and cancer progression when present at sublethal levels. Thus, iron exerts a dual role in cancer biology, by promoting carcinogenesis and tumour growth under certain conditions, while in many cases inducing ferroptotic cell death when oxidative damage exceeds the buffering capacity of cellular defence systems [78]. This balance is affected by intracellular iron distribution, redox status, and lipid composition, all of which may represent potential targets for therapeutic intervention (Figure 2).

Many areas are under investigation in relation to ferroptosis and cancer, including clinical trials involving mechanisms in different cancer cell types and stages, the role of iron and lipid metabolism, the effects of drugs and nutrients, the prospect for anticancer targeting, and the design of new therapeutic strategies. In particular, the possible development and application of chelating and other drugs in anticancer strategies related to ferroptosis could be considered in cancer cell types for which existing therapies are ineffective, as well as in cases of cancer metastasis and drug resistance.

## 4. Variability in the Iron Metabolism–Ferroptosis–Cancer Axis

There are many complexities and variations in the mechanisms and pathways involved in different types and stages of cancer. In each case, the choice of any anticancer therapeutic strategy, including those involving targeting the axis of iron metabolism, ferroptosis, and cancer, requires thorough diagnostic evaluation and “malignancy potential ranking” of all contributory factors and parameters involved. This approach increases the prospects for prioritisation of specific targets of major impact, as well as positive outcomes of such therapeutic strategies, which could be vital for the treatment of many cancers. Furthermore, such information will be especially needed for the design of therapeutic targeting protocols using different approaches for each type and stage of cancer and for each patient, within the context of personalised medicine.

Despite the general involvement of iron in many aspects of cancer malignancy, it seems that newly discovered mechanisms, pathways, and factors identified in some cancers do not follow the general patterns described in the majority of cancer cases, including those described for the general axis of iron metabolism, ferroptosis, and cancer [21,23,26,32]. For example, macrophage ferroptosis has recently been shown to be involved in the pathogenesis and progression of various haematological and other cancers, as well as other diseases [79,80,81]. Similarly, iron overload has been identified in cancer cells and the TME in renal cell carcinoma (RCC). In this case, different cancer evolution patterns were observed, where instead of high iron being implicated during all phases of cancer evolution, iron appears to be primarily involved in early carcinogenesis and early tumourigenesis, but not in later tumour progression. Furthermore, it has also been suggested that iron chelators such as DF and DFRA, which remove iron released by macrophages, inhibit the promotion of tumour growth in RCC [82,83,84].

The control of gene expression, as well as the production of key proteins and other factors involved in iron metabolism, is another area contributing to cancer type variability, progression, and tumour growth. In addition to variations in ferritin (e.g., FTH) expression in different immune cells affecting immune function in relation to the TME and cancer progression described above, changes are also observed in serum ferritin, which contains little or no iron. In particular, increases in serum ferritin levels are of negative prognostic value and are associated with poor prognosis in several cancers, including RCC, breast cancer, prostate cancer, metastatic colorectal cancer, and neuroblastomas [26]. It should be noted that under normal conditions, serum ferritin reflects liver and general body iron stores but is elevated in inflammation and cancer, and it could also be misleading in other conditions [85,86,87]. It is likely that high serum ferritin levels in inflammatory disease are due to increased ferritin secretion by macrophages of the reticuloendothelial system, whereas in cancer, they may be due to increased ferritin secretion by macrophages of the TME.

Another important marker related to iron metabolism is the level of transferrin receptor (TrR) expression on the cell membrane of different types of normal and cancer cells. In particular, elevation of TrR expression is of significant diagnostic and prognostic value, especially in metastatic tumours, where it is associated with poor prognosis [88,89]. For example, in a related study, increases in TrR levels were identified in 12 out of a total of 33 tumour types compared to their respective normal tissues [88,90]. It should be noted that many of the most common cancers, including breast and prostate cancers and also leukaemia, are among the cancer types with increased TrR [28,88,91,92].

The level and function of ferroportin in M2 macrophages of the TME and in tumour cells, both of which are determining factors in iron release, also appear to affect tumour growth and proliferation. For example, in a large study of breast cancer patients, it has been shown that ferroportin levels in human tumours were inversely correlated with malignant potential and clinical outcome [55]. In contrast, it was suggested as a probability that a mutation with loss of function of ferroportin in one patient did not promote cancer cell growth [50]. However, further data and statistical evaluation are required to validate such possibilities. Overall, it seems that the role of ferroportin, and possibly other proteins involved in iron metabolism affecting iron availability in the TME, plays an important role in the growth and malignancy of tumours. Similarly, the role of genomic, transcriptomic, and other parameters of all proteins involved in iron metabolism may affect tumour growth and malignancy [21].

There are many other external and internal factors affecting the variability in the iron metabolism, ferroptosis, and cancer axis. For example, other contributory factors, such as the effects and mechanisms of intestinal flora in the modulation of ferroptosis in immune and other cells, have not yet been fully investigated [93,94]. This information is particularly important for many cancers, especially since the specific pathways involved in the inhibition or promotion of ferroptosis in colitis-associated cancer in inflammatory bowel disease or in haematological cancers have not yet been clearly clarified, and related future investigations may help in the design of target-specific therapeutic strategies [21,79,81].

## 5. Ferroptosis and Cancer Metastasis

Metastasis is the main cause of cancer-related deaths [17,18,19,20,95]. In recent years, ferroptosis has emerged as an important factor in this process, mainly as a context-dependent suppressive mechanism. Its role, however, is not straightforward. In some cancers, ferroptosis acts as a barrier that limits the spread of tumour cells. In others, certain aspects of ferroptosis signalling may actually support metastatic progression. The outcome depends on the cancer type, the surrounding environment, and the molecular features of the tumour cells. Therefore, ferroptosis primarily represents a therapeutic vulnerability in cancer; however, under specific conditions of sublethal oxidative stress or incomplete ferroptotic signalling, ferroptosis-associated pathways may be exploited to support tumour adaptation, inflammation, or progression. Understanding this complexity is essential so that ferroptosis-based therapies can be used to prevent or treat metastatic disease.

### 5.1. Ferroptosis as a Barrier to Metastasis

For tumour cells to metastasise, they must survive several stressful steps. These include detaching from the primary tumour, travelling through the bloodstream, and adapting to a new organ. Metastatic colonisation also requires survival signals from the surrounding stroma cells. Each step exposes cells to oxidative stress and lipid damage. In many cases, ferroptosis limits the survival of cells during this journey. Some examples are outlined below.

In breast cancer, especially in the aggressive TNBC, metastatic cells rely heavily on antioxidant defences to survive [96,97]. Moderately high levels of SLC7A11 and GPX4 contribute directly to protection against lipid peroxidation and ferroptotic cell death in metastatic breast cancer cells, thus conferring a selective survival advantage [98,99]. Thereby, when SLC7A11 and/or GPX4 are experimentally inhibited, metastatic spread decreases both in vitro and in mouse models [97,100,101]. The role of glutamine synthetase, however, is more context-dependent, because although its activity alters intracellular glutamate availability, recent evidence proposes that, under conditions such as glutamine deprivation, glutamine synthetase supports metabolic adaptation and redox homeostasis, thereby shielding metastatic TNBC cells from ferroptosis [102]. This suggests that ferroptosis normally acts as a barrier to metastasis in this setting.

The TME also plays a role. In breast cancer, CAFs can release metabolites, such as glutamine, that contribute to GSH synthesis, and exosomal microRNA [103,104]. These components strengthen antioxidant defences in cancer cells and help them avoid ferroptosis. This shows that stromal cells can indirectly support metastatic cell survival by protecting them from iron-driven lipid damage [105,106].

There are also other examples. As melanoma cells become metastatic, they often shift toward a more mesenchymal or invasive state. This change is linked to alterations in lipid metabolism, including higher incorporation of PUFAs into cell membranes. While these lipid changes may support invasion and migration, they also make the cells more sensitive to lipid peroxidation and ferroptosis [107,108,109]. In different melanoma models, inducing ferroptosis has been shown to reduce lung and ocular metastases [110,111,112,113]. However, metastatic melanoma cells can adapt to this vulnerability. For example, they increase the expression of aldo-keto reductases, which help detoxify lipid peroxidation products and protect against ferroptosis [114]. Other protective mechanisms include upregulation of ferroptosis suppressor protein 1 (FSP1) or secretion of apolipoprotein E (ApoE), both of which reduce sensitivity to ferroptotic stress [115,116], as well as sterol regulatory element-binding protein 2 (SREBP2)-driven transferrin expression that supports iron handling and suppresses ferroptosis in circulating melanoma cells [117]. The role of ApoE was also corroborated in papillary thyroid carcinoma [118]. Together, these findings suggest that although metastatic cells are prone to iron-driven lipid damage, they rely on strong antioxidant and ferroptosis-defence pathways to survive. Targeting multiple protective systems, such as GPX4 and FSP1, may, therefore, be a promising way to limit metastatic spread.

A similar link between epithelial–mesenchymal transition (EMT)-related signalling and ferroptosis sensitivity has been reported in hepatocellular carcinoma, where transforming growth factor-β1 represses SLC7A11 expression and increases vulnerability to GPX4 inhibition. This suggests that EMT-associated transitions may create ferroptotic stress that metastatic cells must overcome [119].

In gastric and colorectal cancers, the situation can depend on the metastatic site. When colorectal cancer (CRC) cells during their metastasis reach the liver, they encounter an iron-rich environment that can promote ferroptosis. Cells that successfully colonise, adapt by enhancing iron and redox homeostasis, such as upregulating antioxidant systems like SLC7A11 to suppress lipid peroxidation and avoid ferroptotic death [120]. CAF-derived exosomes have also been reported to suppress ferroptosis and promote metastatic progression in CRC. For example, exosomal METTL3 (methyltransferase-like 3) from CAFs inhibits ferroptosis by modifying ACSL3 (acyl-CoA synthetase long-chain family member 3) and increases metastasis of CRC cells in vivo, highlighting how stromal cells can modulate ferroptotic responses to support dissemination [121]. Experimentally, increasing iron promotes ferroptosis and limits CRC proliferation and metastasis [122,123]. In gastric cancer models, ferroptosis limits metastatic colonisation [124,125], and inducing ferroptosis through agents acting on the SLC7A11/GPX4 axis, such as dioscin or brucine, has been shown to limit cell migration and invasion, implicating ferroptosis as a barrier to metastatic dissemination [126,127].

Findings in RCC further highlight how changes in iron metabolism may accompany disease progression, as briefly discussed before. In a recent study, Greene et al. [82] showed that iron accumulation is prominent in early-stage RCC but decreases as tumours progress, particularly in sarcomatoid and metastatic lesions. Because ferroptosis depends on iron, this reduction in iron levels during advanced disease may reflect an adaptive strategy that helps tumour cells reduce their susceptibility to ferroptotic death. Although the study did not directly assess ferroptosis, the results support the idea that metastatic progression may involve metabolic adjustments that limit iron-driven lipid damage and enhance tumour cell survival.

**Figure 3:**
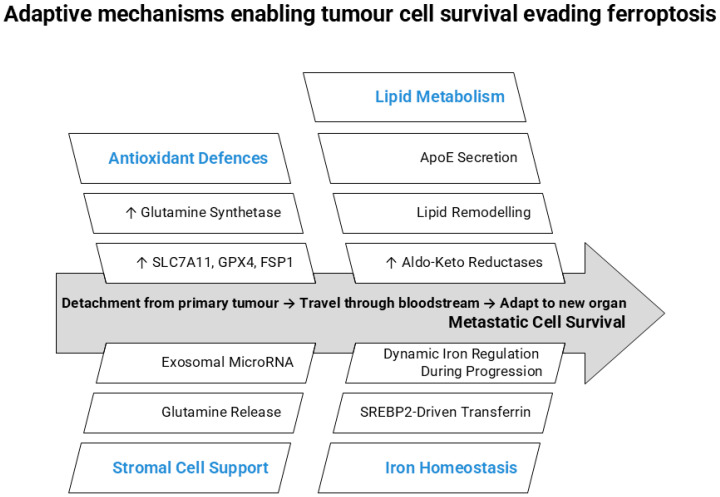
Ferroptosis as a barrier to metastatic progression and adaptive mechanisms that enable tumour cell survival. Ferroptosis appears to act as a barrier to metastatic progression, and several adaptive mechanisms can enable tumour cell survival. During metastasis, which is characterised by tumour cell detachment from the primary tumour, circulation through the bloodstream, and colonisation of distant organs, tumour cells experience oxidative and metabolic stress that can trigger ferroptotic cell death. To overcome this barrier, metastatic cells activate antioxidant defences (e.g., increased glutamine synthetase and upregulation of SLC7A11, GPX4, and FSP1), lipid metabolic adaptations (ApoE secretion, lipid remodelling, and increased aldo-keto reductases), and microenvironmental support mechanisms, including exosomal microRNA signalling and glutamine release. Dynamic regulation of iron metabolism, including SREBP2-driven transferrin expression, further promotes metastatic cell survival. Abbreviations: ApoE: Apolipoprotein E, GPX4: Glutathione Peroxidase 4, FSP1: Ferroptosis Suppressor Protein 1, SREBP2: Sterol Regulatory Element-Binding Protein 2. Arrows indicate relative changes (↑ increase, → causative or regulatory relationship).

Together, these findings suggest that metastatic progression often involves metabolic adaptations that reduce ferroptotic vulnerability (Figure 3).

### 5.2. Ferroptosis as a Potential Promoter of Metastasis

While ferroptosis primarily acts as a barrier to metastasis, emerging evidence suggests that sublethal ferroptosis-associated stress or incomplete ferroptotic signalling, rather than execution of ferroptotic cell death itself, may, under specific conditions, promote tumour adaptation, inflammation, and metastatic progression.

In pancreatic ductal adenocarcinoma (PDAC), tumour cells grow in a dense environment poor in nutrients. That imposes significant metabolic stress. Such microenvironmental stresses are generally known to activate epithelial–mesenchymal plasticity and related stress-response pathways, which can enhance cellular plasticity and migration [128]. In pancreatic cancer cells, nutrient deprivation has been shown to modulate mesenchymal state and ferroptosis sensitivity through extracellular signal-regulated kinase 1/2 (ERK1/2) and c-Jun N-terminal kinase (JNK) signalling [129]. Lipid peroxidation products such as 4-hydroxynonenal (4-HNE) generated during ferroptotic lipid damage can activate transcription factors such as nuclear factor erythroid 2-related factor 2 (NRF2) and activator protein-1 (AP-1) [130,131,132]. These factors promote genes linked to survival, inflammation, and motility, which may support invasion and metastasis in cancer contexts. For example, in a Kirsten rat sarcoma virus oncogene homolog (KRAS)-driven pancreatic cancer model, GPX4 depletion or a high-iron diet enhanced ferroptotic stress and significantly increased tumour invasion and metastasis to the liver and lung, mediated through STING-dependent inflammatory signalling [133]. These findings demonstrate that ferroptosis-associated damage and stress could create a pro-tumourigenic microenvironment that could facilitate metastatic progression; thus, targeting ferroptosis in pancreatic cancer could prove a double-edged sword [134,135].

A similar concept has recently been suggested in non-small cell lung cancer (NSCLC), where mechanisms that confer resistance to ferroptosis, such as SNHG3 (small nucleolar RNA host gene 3) hyper-editing, can promote metastatic activity and invasiveness [136]. However, direct evidence that ferroptotic activation promotes metastasis is limited so far, although alterations in ferroptosis regulatory pathways, particularly those leading to ferroptosis resistance, have been hypothesised to be associated with increased metastatic potential in some experimental models.

### 5.3. Organ-Specific Effects of Ferroptosis in Metastasis

The impact of ferroptosis on metastasis also depends on the organ involved. For example, in brain metastasis, usually derived from breast or lung cancers, tumour cells must adapt to an environment rich in lipids but with limited antioxidant buffering. Metastatic cells that successfully colonise the brain often increase lipid-detoxifying enzymes and mitochondrial antioxidant defences [137,138]. These changes could help them avoid ferroptotic death, as well as become resistant to drugs that affect the cellular redox balance [139]. Targeting these adaptations could make brain metastases more sensitive to ferroptosis [140].

Bone metastases present a different challenge. Emerging evidence suggests that alterations in iron metabolism within the bone metastatic niche, which may arise in part from tumour-driven modulation of iron handling and increased availability of redox-active iron, lead to increased iron accumulation within bone and bone marrow. This promotes oxidative stress, osteoblast suppression, and enhanced osteoclast activity, ultimately driving bone loss and destruction of the microarchitecture. Conversely, iron deficiency, including in the context of cancer-associated anaemia and inflammation-induced iron sequestration, also impairs bone remodelling, indicating that tightly regulated iron homeostasis is critical for skeletal integrity, and that any imbalance may be particularly important in the ferroptosis-relevant microenvironment of bone metastases [141,142,143,144,145,146,147,148,149,150]. In breast cancer bone metastasis, metastatic cells exploit the bone microenvironment by engaging niche macrophages that recycle iron and increase iron availability within the metastatic niche, promoting tumour growth. This iron enrichment increases the LIP and may enhance susceptibility to ferroptosis, suggesting that disrupting iron homeostasis or antioxidant defences could selectively target breast cancer cells metastasising to the bone [55,151]. Similarly, prostate cancer cells upregulate iron uptake and downregulate iron export to preserve a high LIP that supports proliferation and metastatic behaviour [152]. Although direct studies on bone-specific iron dysregulation are limited, preclinical evidence suggests that prostate cancer cells metastasising to the bone remain susceptible to ferroptosis induction and that disrupting antioxidant defences or enhancing iron availability may antagonise metastatic growth [153,154,155].

## 6. Ferroptosis and Drug Resistance Mechanisms

Resistance to chemotherapy and targeted therapies is a major reason why many cancers fail to respond or eventually relapse after treatment. In the last few years, research has shown that ferroptosis is closely linked with drug resistance. In many cases, cancer cells that avoid ferroptosis become resistant to standard therapies. In other cases, inducing ferroptosis helps overcome resistance and restore drug sensitivity [156].

One of the core ways cancer cells become resistant is by upregulating antioxidant defences that suppress ferroptosis. For example, many drug-resistant tumours show high activity of GPX4 and NRF2, both of which limit lipid peroxidation and ferroptotic cell death. High GPX4 expression has been correlated with resistance to platinum-based chemotherapy in several tumours, because it neutralises lipid peroxides that would otherwise trigger ferroptosis when cells are stressed by drugs [139,157,158]. Similarly, NRF2 activation increases antioxidant capacity and reduces ferroptosis susceptibility, helping cancer cells survive under chemotherapeutic treatments [159,160].

There are many tumour-specific examples implicating ferroptosis to drug resistance. For example, in digestive cancers, such as gastric and CRC, evidence shows that resistance to widely used drugs (like cisplatin, oxaliplatin, and other targeted agents) is linked to impaired ferroptosis. In gastric cancer models, suppressing the system X_c_^−^ transporter (comprising cystine transporter SLC7A11 or xCT) or GPX4, increases lipid peroxidation and reverses resistance to cisplatin and sorafenib, as well as radioresistance, by restoring ferroptotic cell death pathways [161,162,163]. In CRC, combining ferroptosis inducers with anti-EGFR (epidermal growth factor receptor) therapy enhances the efficacy of treatments that otherwise fail in rat sarcoma (RAS)-mutant tumours, in part by downregulating antioxidant responses and promoting ferroptosis [164].

In ovarian cancer, particularly in tumours treated with poly(ADP-ribose) polymerase (PARP) inhibitors such as olaparib, recent preclinical studies show that PARP inhibition primes ovarian cancer cells for ferroptosis, while induction of ferroptosis significantly enhances tumour cell killing and can overcome acquired resistance mechanisms [165,166,167]. This suggests that ferroptosis modulation can help overcome resistance in targeted therapy settings.

Ferroptosis also links with resistance in other solid tumours. In some resistant cancers, such as head and neck and lung cancers, elevated expression of FSP1, which protects cells independently of GPX4, has been linked to reduced sensitivity to drugs inducing ferroptosis. In breast cancer, resistance has similarly been associated with enhanced activity of the xCT–GPX4 antioxidant axis. In these contexts, targeting GPX4-dependent pathways or inhibiting FSP1 appears to make resistant cells more vulnerable to ferroptosis and may help overcome drug resistance [168,169,170,171]. In hepatocellular carcinoma, cells that evade ferroptosis show increased survival after sorafenib or other tyrosine kinase inhibitors, while combining these drugs with ferroptosis inducers can resensitise resistant cells by promoting iron-mediated lipid peroxidation [172,173,174].

Another pattern emerging across cancer types is that changes in iron metabolism itself contribute to resistance. Resistant cancer cells often modulate their LIP or increase iron export mechanisms or alter iron–sulfur cluster and mitochondrial iron regulation in ways that suppress ferroptosis and allow survival under drug treatment [175,176,177,178]. Conversely, strategies that increase intracellular iron, for example by disrupting ferritin storage or promoting iron import, can promote ferroptosis and restore drug sensitivity in resistant cancer cells [21,23,179].

Recent evidence also shows that ferroptosis resistance contributes to cancer cell survival under immune pressure, linking ferroptosis to resistance mechanisms beyond conventional drug treatment. A recent study demonstrated that cancer cells can actively evade ferroptosis induced by cytotoxic immune cells by upregulating fatty acid binding proteins (FABPs), which limit the availability of lipids that can be peroxidised in cellular membranes [180]. By sequestering PUFAs and reducing lipid peroxidation, this mechanism suppresses iron-dependent oxidative damage and prevents ferroptotic cell death, even in the presence of strong oxidative stress mediated by the immune system.

In addition to synthetic agents, several natural compounds have been shown to modulate ferroptosis through effects on iron metabolism, lipid peroxidation, and antioxidant defence systems. Polyphenols such as quercetin, curcumin, and fisetin can influence ferroptosis sensitivity by altering intracellular redox balance, modulating GPX4 activity, and affecting iron availability in a context-dependent manner [181,182,183]. High-dose ascorbic acid has also been reported to promote iron-dependent oxidative stress and sensitise cancer cells to ferroptosis under specific conditions [184,185]. Importantly, these compounds may be particularly relevant in combination strategies, where they could lower the ferroptosis threshold and enhance the efficacy of conventional chemotherapy, radiotherapy, or targeted agents by sensitising tumours to ferroptosis, rather than acting as monotherapies.

**Figure 4:**
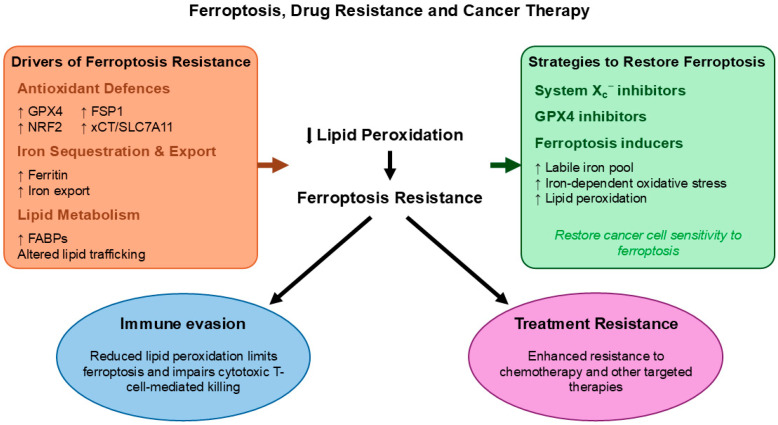
Ferroptosis resistance as a driver of drug resistance and a potential therapeutic target in cancer. Several adaptive mechanisms reduce lipid peroxidation and allow cancer cells to evade ferroptotic cell death. These include increased antioxidant defences (e.g., GPX4, NRF2, FSP1, and SLC7A11), which limit oxidative damage, and changes in iron metabolism, such as increased ferritin expression and iron export, which reduce the labile iron pool and suppress iron-dependent ROS generation. In addition, alterations in lipid metabolism, including increased FABPs, reshape lipid composition and decrease the availability of peroxidation-prone lipids, further lowering ferroptosis sensitivity. Together, these changes lead to reduced lipid peroxidation, resulting in ferroptosis resistance and ultimately contributing to treatment resistance. Reduced ferroptotic activity also supports immune evasion, as lower lipid peroxidation can impair cytotoxic T cell-mediated killing of cancer cells. To counteract these effects, therapeutic strategies aim to restore ferroptosis. These include GPX4 inhibitors, system X_c_^−^ inhibitors, and approaches that increase intracellular iron and oxidative stress. By promoting lipid peroxidation and ferroptotic cell death, these interventions can help overcome resistance and improve treatment responses. Abbreviations: xCT: Cystine/Glutamate Antiporter, GPX4: Glutathione Peroxidase 4, NRF2: Nuclear Factor Erythroid 2-Related Factor 2, FSP1: Ferroptosis Suppressor Protein 1, FABPs: Fatty Acid Binding Proteins. Arrows indicate relative changes (↑ increase, ↓ decrease, → causative or regulatory relationship).

In summary, accumulating evidence shows that ferroptosis resistance is a common feature of many drugs or other therapies in resistant cancers. Targeting ferroptosis pathways by inhibiting GPX4 and other antioxidant defences, altering iron metabolism, or combining ferroptosis inducers with existing drugs has emerged as a promising strategy to overcome treatment resistance across cancer types (Figure 4). Further research into precisely how ferroptosis interacts with traditional therapy resistance mechanisms could lead to the development of new combination treatments that can improve patient outcomes [186].

A summary of how ferroptosis is involved in different cancer types and how it affects treatment response is shown in Table 1. Across tumours, common patterns emerge, including changes in iron metabolism, redox balance, lipid composition, and antioxidant defences such as the SLC7A11/GPX4 pathway. Some cancers, including non-small cell lung cancer, glioblastoma, and hepatocellular carcinoma, appear particularly sensitive to lipid peroxidation driven by iron. Others develop resistance by strengthening antioxidant systems, altering lipid metabolism, for example, by increasing monounsaturated fatty acids, or enhancing iron export. These adaptations influence how tumours respond to chemotherapy, targeted therapy, and immunotherapy. Overall, ferroptosis emerges as an important metabolic vulnerability in cancer, while its successful regulation depends on the specific tumour context.

## 7. Therapeutic Targeting of Iron Metabolism and Ferroptosis in Cancer

There are many targeting options for anticancer therapeutic strategy interventions based on ferroptosis, including the targeting of associated proteins, transcription factors, iron metabolic pathways, lipid metabolic pathways, free radical generation and associated metabolic pathways, redox iron, ferritinophagy, macrophage-stored iron, and many others [21,22,38,39,72,230,231]. In this context, the control of iron availability and related processes in cancer cells and the TME could have a major impact on anticancer therapeutic strategies.

Iron, iron-containing proteins, iron metabolism, and ferroptosis play an important role in malignancy, immune evasion, and drug resistance in cancers. In particular, iron-laden TAMs, characterised by excess iron deposition in the form of hemosiderin, are a major feature in several cancers, including breast and prostate cancer [21,232,233,234,235]. This excess iron, detectable by magnetic resonance imaging (MRI) and other magnetic resonance spectroscopic techniques, is associated with tumour growth, metastasis, and reduced efficacy of therapeutic interventions, including immunotherapy. In contrast, iron chelators, and especially L1, can efficiently remove iron from macrophages and reverse these processes [232,233,234,235,236,237,238].

### 7.1. Iron Chelation Therapy in Oncology

Iron chelation therapy represents a key strategy for modulating iron dysregulation and ferroptosis in cancer [21]. Different chelation protocols can be tailored for each cancer patient based on risk/benefit assessment and individual characteristics in the context of personalised medicine, as previously applied in thalassemia and myelodysplasia patients [239,240,241,242,243,244,245]. For example, intensive chelation protocols combining L1 and DF may be used in non-anaemic patients with RCC and other cancers with excess iron load, whereas lower doses of L1 may be more appropriate in anaemic cancer patients, who are presented with excess iron accumulation in the TME [236,237,239,240,241]. Monitoring of iron parameters, such as with the use of MRI and other techniques, alongside toxicity and disease indicators, are essential for evaluating the efficacy and safety of these protocols, including when used in combination with chemotherapy, radiotherapy, or other treatments [239,240,241,242,243,244,245,246].

The three main iron-chelating drugs, L1, DFRA, and DF, are widely used in iron overload conditions, including some cancer-related settings, although their systematic evaluation as anticancer agents, particularly in combination therapies, remains limited [247]. Their clinical use requires careful risk/benefit assessment due to potential toxicity, especially in patients with normal or low iron levels, where DFRA and DF are generally not recommended [248]. Nevertheless, extensive data on their safety and efficacy in other patient populations with physiological levels of iron load, and especially the wide use of L1 in such patient categories, support the potential repurposing of iron-chelating drugs in oncology [236,237,238,239,240,241,242,243,244,245,246,247,248].

### 7.2. Molecular and Cellular Mechanisms of Iron-Chelating Agents

**Figure 5:**
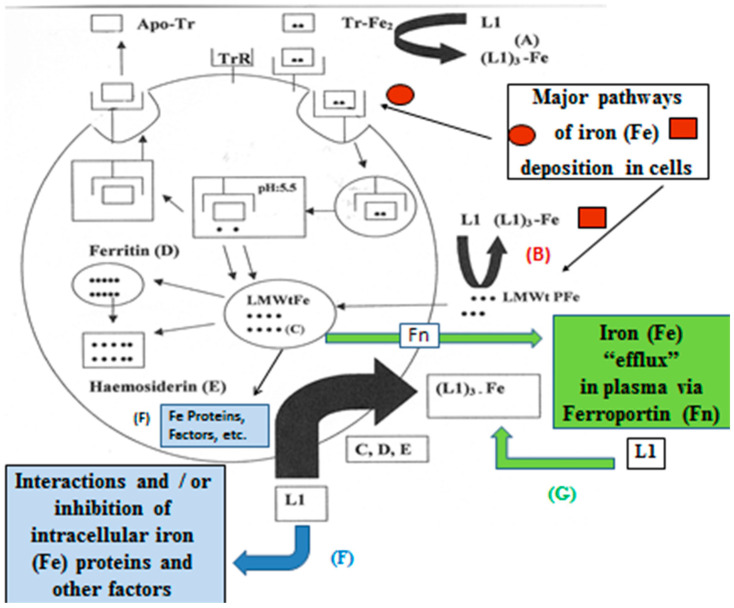
Iron mobilisation from the iron pools of a cancer cell and plasma by deferiprone (L1). The schematic illustration shows the general iron loading process of cells and the mode of action of transferrin (Tr) iron deposition via a transferrin receptor (TrR), and also non-transferrin-bound iron. Deferiprone may prevent iron accumulation in cells through iron removal from transferrin in plasma (A), the low-molecular-weight plasma iron pool (LMWtPFe) (B), and iron efflux from the cell via ferroportin (Fn) (G) (in green). Deferiprone may also mobilise iron from the intracellular low-molecular-weight iron pool (LMWtFe) (C), ferritin (D), and hemosiderin (E) and inhibit the turnover of iron proteins and other factors (F) (in blue). Iron removal by L1 from the A–D iron pools is shown in broad black arrows. Abbreviations: Fn: Ferroportin, L1: Deferiprone, LMWtFe: Low-molecular-weight iron pool, LMWtPFe: Low-molecular-weight plasma iron pool, Tr: Transferrin, TrR: Transferrin receptor, Apo-Tr: Apotransferrin (transferrin not bound to iron).

At the molecular level, the higher iron requirements of cancer cells render them particularly vulnerable to iron depletion. Deferiprone has been shown to mobilise iron in a concentration-dependent manner from multiple pools, including transferrin, lactoferrin, ferritin, hemosiderin, the low-molecular-weight intracellular iron pool (LMWtFe), and plasma iron pools (LMWtPFe), as well as iron released via ferroportin [248,249] (Figure 5). The mobilisation of iron from a wide range of iron pools from cancer cells and TME-associated cells is likely to reduce tumour growth, malignancy, immune evasion, metastasis, and drug resistance.

Additional anticancer effects of L1 and other chelators include inhibition of iron-dependent enzymes such as ribonucleotide reductase, aconitase, lipoxygenase, cyclooxygenase, and hydroxylases, as well as modulation of transcription factors including hypoxia-inducible factor (HIF), the metastatic suppressor N-MYC downstream-regulated gene-1 (NDRG1), and STEAP4 (six-transmembrane epithelial antigen of prostate 4) metalloreductase, all of which are involved in tumour proliferation and metastasis [21,249]. Other mechanisms include the inhibition of prolyl-4-hydroxylase, which causes a reduction in collagen synthesis required for tumour progression and CAF activity [21,249,250,251], and the targeting of iron-dependent histone lysine demethylases, resulting in epigenetic changes with anti-tumour effects [252]. Furthermore, L1 has demonstrated potent activity against cancer stem cells by targeting mitochondrial metabolism and has been proposed for phase II clinical trials in breast and other cancers [253]. An additional important advantage of L1 over the other chelating drugs is its ability to cross the blood/brain barrier and potentially be used for brain tumour therapies [21].

### 7.3. Modulation of Ferroptosis by Iron-Chelating Drugs

The modulation of iron metabolism, ferroptosis, and the cancer axis for therapeutic purposes using chelating and other drugs is complex. Although iron-chelating drugs and other chelators appear in general to inhibit ferroptosis, other targeted molecules and mechanisms may prevail, resulting in the opposite effect. Importantly, iron chelation does not aim to activate ferroptosis directly but rather to suppress tumour-promoting iron-dependent metabolic and oxidative processes. In this context, iron chelation and ferroptosis induction represent complementary but mechanistically distinct therapeutic strategies, whose application must be guided by tumour iron status, redox balance, and diagnostic biomarkers, as already discussed.

Iron-chelating drugs can influence ferroptotic mechanisms in multiple ways. All three chelating drugs inhibit iron-catalysed free radical reactions, while L1 and DF also inhibit iron-dependent enzymes such as cyclooxygenase and lipoxygenase involved in lipid peroxidation and ferroptosis [21,22,249]. Conversely, under specific conditions, L1 can induce ferroptosis through modulation of oncogenic pathways such as the magnesium transporter cyclin M4 (CNNM4) expression in cholangiocarcinoma cells [254]. This dual role highlights the complexity of targeting iron metabolism and ferroptosis in cancer.

Overall, numerous studies indicate that the management of iron metabolism and ferroptosis could play a pivotal role in anticancer therapeutic strategies. Advances in the diagnosis of iron distribution in tumours and the TME, along with the development of personalised iron chelation protocols, may enable their integration into clinical practice either as monotherapies or in combinations with existing therapies. Ultimately, anticancer strategies should be guided by diagnostic criteria assessing the relevance of specific targets, including iron metabolism and ferroptosis pathways, in each cancer type and stage.

## 8. Clinical Translation of Ferroptosis-Based Therapies

Clinical translation of ferroptosis modulation in cancer remains at an early but rapidly evolving stage. Initial human studies demonstrate that ferroptosis can be manipulated through three strategies: iron delivery, redox pathway inhibition, and metabolic reprogramming, with increasing integration of biomarkers for the inclusion of patients. Some examples of recent or ongoing clinical studies are described below.

### 8.1. Ferroptosis-Inducing and Modulating Agents in Clinical Trials

Direct ferroptosis induction via iron loading has entered early clinical trials. Phase I trials of intratumoural carbon nanoparticle-loaded Fe(II) (CNSI-Fe) in advanced solid tumours (clinical trials NCT06048367 and NCT07433283) established feasibility, manageable safety profiles, and absence of dose-limiting systemic iron toxicity. Although efficacy and long-term toxicity data remain preliminary, these studies provide proof-of-concept that a specific and controlled increase in tumour iron can be clinically applied to promote oxidative lipid damage to advanced solid tumours. Other agents capable of amplifying iron-dependent oxidative stress are also being explored clinically. For example, artesunate, originally developed as an antimalarial drug [255], has entered early-phase clinical trials in breast cancer and other solid tumours (e.g., NCT03792516), where its ability to increase intracellular iron levels and ROS may contribute to ferroptosis induction.

The targeting of cystine metabolism and antioxidant defences is also a clinically accessible approach. Repurposed agents such as sulfasalazine, a SLC7A11/system X_c_^−^ inhibitor, have been evaluated in glioma, alone or in combination with stereotactic radiosurgery (indicative clinical trials NCT01577966, NCT04205357) [256]. Similarly, sorafenib, a multi-kinase inhibitor (RAF, VEGFR, PDGFR) that also targets SLC7A11, has been shown to sensitise colorectal liver metastases to radiotherapy [257]. Several other clinically used drugs may influence ferroptosis through related redox mechanisms. For instance, buthionine sulfoximine, which inhibits GSH synthesis by inhibition of γ-glutamylcysteine synthetase, has been evaluated in early-phase trials in neuroblastoma (e.g., NCT00002730), where depletion of intracellular GSH lowers GPX4 activity and enhances lipid peroxidation. Classical cytotoxic agents such as cisplatin, a DNA cross-linker and adduct formator, may also contribute to ferroptotic cell death by depleting GSH and impairing GPX activity [258,259], thereby weakening antioxidant defence systems. These studies support the idea that ferroptosis induction may function most effectively as a radiosensitising or chemosensitising strategy, rather than as monotherapy.

Furthermore, emerging data in acute myeloid leukaemia indicate that metabolic remodelling, including alterations in fatty acid metabolism, can lower the threshold to induce ferroptosis. The changes in lipid metabolism by imetelstat, primarily a telomerase inhibitor [260] and similar combination regimens, e.g., MLN4924 with belinostat, a histone deacetylase inhibitor [261], show how ferroptosis susceptibility may be increased indirectly through disruption of metabolic and other networks. In addition, pharmacological modulation of the mevalonate pathway by statins such as fluvastatin or simvastatin, which are under investigation in several cancer trials (e.g., NCT00416403, NCT01992042, NCT00313859, NCT00944463), may influence ferroptosis sensitivity by reducing isoprenoid synthesis and thereby affecting GPX4 activity and cellular lipid peroxide handling. This suggests that the idea that lipid metabolism affects ferroptosis sensitivity is not limited to cancer models. A randomised crossover dietary study in healthy individuals showed that altering fatty acid composition can increase ferroptosis in human colonocytes, highlighting that metabolic regulation of ferroptosis is achievable in vivo [262].

For selected ferroptosis-related agents, indicative clinical trials, clinical phase or approval status in oncology, tested cancer types, and the proposed mechanism linking the drug to ferroptosis regulation are summarised in Table 2. Many of these studies were not originally designed to specifically target ferroptosis but provide clinical context for pharmacological strategies that may modulate ferroptotic susceptibility in tumours.

Beyond pharmacological agents, several nutraceutical compounds with redox-modulating properties are also being explored in clinical settings and may influence ferroptotic pathways [247]. Compounds such as quercetin, curcumin, fisetin, and high-dose ascorbic acid are currently being evaluated in early phase trials across a range of malignancies, including breast cancer, glioma, and CRC. Many of these compounds have been shown experimentally to modulate GPX4 activity, GSH levels, or intracellular iron handling, thereby promoting lipid peroxidation and ferroptosis under certain conditions [181,182,183,184,185,247,263,264].

### 8.2. Iron-Chelating Agents as Anti-Ferroptotic Modulators in Clinical Trials

A number of clinically used drugs can inhibit ferroptosis through iron chelation or antioxidant effects, highlighting the importance of iron metabolism in modulating ferroptotic susceptibility. In this context, the iron-chelating drugs DF, DFRA, and L1 have been shown to bind free iron and reduce the availability of redox-active iron required for lipid peroxidation [247,248], while compounds such as N-acetylcysteine (NAC) replenish intracellular thiols and GSH levels. These agents are currently being investigated in clinical trials in several malignancies, including metastatic TNBC and acute leukaemia, and illustrate the dual role of iron metabolism modulation in either promoting or limiting ferroptotic cell death depending on therapeutic context (Table 3).

### 8.3. Biomarkers and Patient Stratification for Ferroptosis-Based Therapies

In the meantime, genomic analyses in RCC [266], lung adenocarcinoma [267], and hepatocellular carcinoma [268] show that ferroptosis-related lncRNA signatures correlate with prognosis, immune landscape composition, and predicted immunotherapy responsiveness. These findings suggest that ferroptotic pathways may serve not only as therapeutic targets but also as predictive biomarkers, linking iron metabolism, lipid peroxidation sensitivity, and tumour/immune system interactions.

Altogether, current evidence indicates that ferroptosis-based strategies are clinically feasible. However, most studies are still in the early phase, depend on specific biomarkers, and mainly address mechanistic information. Larger randomised trials looking for survival outcomes are still ongoing. Current and future attempts will likely depend on the following:

(i) Combination regimens combining ferroptosis induction with radiotherapy, chemotherapy, or immunotherapy;

(ii) Patient selection based on ferroptosis susceptibility and biomarkers;

(iii) Careful management of systemic oxidative and all other forms of toxicity.

Thus, while ferroptosis has not yet proven an effective monotherapy in cancer, its integration into combinatorial treatments represents a promising strategy for overcoming resistance to existing treatments and exploiting iron-dependent metabolic vulnerabilities in cancer.

## 9. Conclusions 

Almost all cancers and their stages of malignancy exhibit distinct characteristics influenced by multiple interacting factors, including iron toxicity, dysregulated iron-metabolic proteins, and cell-death pathways, including ferroptosis. Of equal importance to tumour malignancy and its targeting is the identification of other factors and special prevalent characteristics, such as the over-expression of different genes, participation of different cells in the TME, such as TAMs, and susceptibility of specific cells, such as cancer stem cells, to various drugs. The diagnoses of the level of impact of these various factors, cells, and characteristics in all different forms of malignancy in each cancer type, including those related to iron metabolism and ferroptosis, are crucial for the design of anticancer strategies with specific targeting, including the preparation of effective therapeutic protocols in the context of personalised medicine.

The prospect of the use of iron-chelating drugs and other modulators of iron and ferroptosis in combination with existing anticancer therapies is another important area of anticancer drug development, which could have a major impact on cancer treatments in the context of personalised medicine, including the possibility that their clinical use could cause the reduction of toxicities of other therapeutic interventions.

## Figures and Tables

**Table 1:** Ferroptosis in different types of cancers: Key mechanisms, biomarkers, and therapeutic implications.

Cancer Type	Key Mechanisms	Indicators/ Biomarkers	Therapeutic Consequences	Indicative References
**Non-Small Cell Lung Cancer**	Irregular iron metabolism High TrR expression increases labile iron ROS accumulation promotes PUFA lipid peroxidation	TrR, ferritin, lipid peroxides	Most NSCLC cells show vulnerability to ferroptosis due to chronic oxidative stress and iron accumulation → potential target for ferroptosis-based therapy Some KRAS-driven tumours upregulate NRF2/GPX4 to gain resistance	[187,188,189,190,191]
**Gastric Cancer**	↑ SLC7A11/GPX4 axis controls cystine uptake and GSH synthesis Transcription regulation (ATF3/ATF4) alters ferroptosis sensitivity Altered ferritin and lipid metabolism affect lipid peroxide accumulation EMT status affects ferroptosis context-dependently	SLC7A11, GPX4, ROS, EMT markers	Gastric tumours use antioxidant pathways to stop ferroptosis → manipulating these pathways could enhance chemosensitivity	[107,192,193,194,195]
**Colorectal Cancer**	↑ SLC7A11/GPX4 axis Modulation by p53/SLC7A11 axis affects ferroptosis and drug response Altered lipid metabolic regulators (ACSL4, ELOVL6) affect sensitivity to lipid peroxidation β-catenin signalling correlates with SLC7A11/GPX4 expression and ferroptosis resistance	SLC7A11, GSH, ROS, GPX4	CRC uses antioxidant defences and metabolic regulation to cause ferroptosis resistance → targeting SLC7A11/GPX4 may reverse chemoresistance	[196,197,198,199,200]
**Glioblastoma**	Changes in redox balance and defensive antioxidant capacity Modulation of GPX4 and lipid peroxidation pathways SIRT3 and STAT3 regulation affect ferroptosis sensitivity Dysregulated iron uptake and ROS generation contribute to ferroptosis regulation	Lipid peroxides, GPX4	Targeting iron and lipid ROS improves therapeutic response in GBM experimentally → ferroptosis induction explored as novel strategy	[201,202,203,204]
**Breast Cancer**	Lipid remodelling (↑ MUFA and ACSL3) alters membrane composition to limit lipid peroxidation → ferroptosis resistance Regulation by noncoding RNAs and other lipid metabolic pathways affects ferroptosis susceptibility	ACSL3, lipid profile, ROS	Breast cancers remodel lipids to evade ferroptosis → targeting lipid metabolism may overcome resistance	[205,206,207,208,209]
**Pancreatic Cancer**	Diet and lipid composition (PUFA/MUFA balance) influence ferroptosis sensitivity High MUFA environments suppress lipid peroxidation	Lipid composition ratios, ROS	PDAC often shows ferroptosis resistance due to altered lipid metabolism → targeting dietary/lipid pathways may experimentally impact drug response	[210,211,212,213,214]
**Ovarian** **Cancer**	Redox dysregulation sensitises cells to iron-dependent lipid damage ↑ SLC7A11/GPX4 axis limits ferroptosis PARP inhibition modulates ferroptosis pathways	SLC7A11, GPX4, ROS	Upregulation of SLC7A11/GPX4 antioxidant axis → chemoresistance Ferroptosis inducers enhance response to PARP inhibitors: BRCA-deficient: GPX4 inhibition enhances PARP inhibitor sensitivity by promoting ferroptosis BRCA-proficient: PARP inhibition suppresses SLC7A11 and synergises with ferroptosis inducers to enhance antitumour responses → Combined approaches using ferroptosis modulators may overcome resistance in ovarian tumours	[157,167,215,216]
**Hepatocellular Carcinoma**	Iron overload promotes lipid ROS ↑ NRF2/FSP1 antioxidant defences impair ferroptosis Interplay with YAP/TAZ signalling to regulate ferroptosis	ROS, NRF2, FSP1	HCC uses antioxidant responses to deal with ferroptosis → modulating these defences can alter tumour growth	[217,218,219,220,221]
**Melanoma**	Altered ROS and lipid peroxidation pathways Lipid handling changes influence ferroptotic responses (e.g., dependence on lipid peroxides) Ferroptosis acts as tumour suppressor via iron-induced lipid peroxidation context-dependently	Lipid peroxides, ROS	Ferroptosis may limit melanoma progression under specific metabolic states Interplay with immune microenvironment is also being explored	[222,223,224,225]
**Haematological Malignancies**	Distinct iron and lipid metabolic regulation due to bone marrow microenvironment High basal oxidative stress and altered redox homeostasis → ↑ ferroptotic sensitivity compared to many solid tumours	LIP, TrR, SLC40A1, ferritin and its mediators, lipid peroxides, ROS, ACSL4	Ferroptosis inducers can synergise with conventional chemotherapies to kill leukaemia or lymphoma cells that are otherwise resistant to apoptosis	[226,227,228,229]

Abbreviations: ACSL3: Acyl-CoA synthetase long-chain family member 3, ACSL4: Acyl-CoA Synthetase long-chain family member 4, ATF3: Activating transcription factor 3, ATF4: Activating transcription factor 4, BRCA: Breast cancer gene, CRC: Colorectal cancer, ELOVL6: Elongation of very long chain fatty acids protein 6, EMT: Epithelial–mesenchymal transition, FSP1: Ferroptosis Suppressor protein 1, GBM: Glioblastoma, GPX4: Glutathione peroxidase 4, GSH: Glutathione, HCC: Hepatocellular carcinoma, KRAS: Kirsten rat sarcoma virus oncogene homolog, LIP: Labile iron pool, MUFA: Monounsaturated fatty acid, ncRNA: Non-coding RNA, NRF2: Nuclear factor-erythroid factor 2-related factor 2, NSCLC: Non-small cell lung cancer, PARP: Poly(ADP-ribose) polymerase, PDAC: Pancreatic ductal adenocarcinoma, PUFA: Polyunsaturated fatty acid, ROS: Reactive oxygen species, SIRT3: Sirtuin 3, SLC7A11: Solute Carrier Family 7 Member 11, SLC40A1: Solute Carrier Family 40 Member 1, STAT3: Signal transducer and activator of transcription 3, TAZ: Transcriptional coactivator with PDZ-binding motif, TrR: Transferrin receptor 1, YAP: Yes-associated protein. Arrows indicate relative changes or directional effects (↑ increase, → causative or regulatory relationship).

**Table 2:** Representative clinical trials of agents modulating ferroptosis in cancer.

Therapeutic Agent	Indicative Clinical Trials	Clinical Phase/Approval Status for Cancer	Tested Cancer Type	Relation to Ferroptosis	Mechanism Related to Ferroptosis
Ferroptosis-Related Agents					
Zalcitabine	NCT00000954	I	AIDS-related Kaposi sarcoma	Mitochondrial DNA maintenance	Impairs mtDNA replication and repair → ↑ oxidative stress
Buthionine Sulfoximine	NCT00002730, NCT00005835	I	Neuroblastoma	Glutathione synthesis inhibition	Blocks γ-GCS → ↓ GSH → ↓ GPX4 → ↑ lipid peroxidation
Altretamine	NCT00002936	I	HIV-related lymphoma and sarcoma	GPX4 inhibition	Disrupts thiol/antioxidant systems (GPX4 inactivation) → ↑ oxidative stress
Cisplatin	NCT01656551, NCT04809103, NCT01097252, NCT03880396, NCT00463788, NCT03275857	Marketed	NSCLC, cervical cancer, squamous cell carcinoma of the head and neck, breast cancer, prostate cancer, etc.	GSH depletion/GPX inactivation	↓ intracellular GSH → ↓ GPX activity
Sorafenib	NCT03794440, NCT03247088, NCT02559778, NCT00064350	Marketed	HCC, AML, NSCLC, neuroblastoma	System X_c_^−^ inhibition	Inhibits system X_c_^−^ → ↓ cystine → ↓ GSH → ↑ oxidative stress sensitises cells to ferroptosis
Sulfasalazine	NCT01577966, NCT04205357, NCT01198145, NCT03847311, NCT05664464, NCT06134388	I, II, III	Glioma, glioblastoma, breast cancer, colorectal cancer, other solid tumours	System X_c_^−^ inhibition	Inhibits system X_c_^−^ → ↓ cystine → ↓ GSH → ↑ oxidative stress sensitises cells to ferroptosis
Lamivudine	NCT03144804, NCT00041327	II	Colorectal cancer, T cell leukemia/lymphoma	SOD1 increase	Activates PGK1 and SOD1 → ↑ antioxidant defences → ↓ lipid peroxidation → ↓ ferroptosis
Temozolomide	NCT00626990, NCT00005597, NCT00740636, NCT00576680	Marketed	Glioma, gastrointestinal tumours, lung cancer, pancreatic cancer	NRF2 / stress response modulation	Induces system X_c_^−^ via NRF2/ATF4 pathways
Fluvastatin	NCT00416403, NCT01992042, NCT02115074, NCT06679036	I, II	Breast cancer, prostate cancer, glioma	Mevalonate pathway inhibition	Blocks mevalonate pathway → ↓ isoprenoids → ↓ GPX4/system X_c_^−^ → altered lipid peroxide handling
Simvastatin	NCT00354640, NCT00281476, NCT03086291, NCT02026583, NCT01099085, NCT04457089, NCT00313859, NCT00944463	I, II, III	Breast cancer, multiple myeloma, colorectal cancer, gastric cancer, ovarian cancer, pancreatic cancer	Mevalonate pathway inhibition	Blocks mevalonate pathway → ↓ isoprenoids → ↓ GPX4/system X_c_^−^ → altered lipid peroxide handling
Artesunate	NCT03792516, NCT02353026, NCT02354534, NCT03100045, NCT00764036, NCT06165614	I	Breast cancer, vulvar intraepithelial neoplasia, cervical intraepithelial neoplasia, anal intraepithelial neoplasia, advanced solid tumours	Iron-dependent ROS amplification	↑ Fe^2+^ and ROS, ↓ GPX4/GSH, disrupts iron regulation → ↑ lipid peroxidation
CNSI-Fe(II) (carbon nanoparticle-loaded iron)	NCT06048367, NCT07433283	I, II	Advanced solid tumours	Targeted iron load	↑ intratumoural iron → ↑ labile Fe^2+^ → lipid peroxidation → ferroptosis
Neratinib	NCT03457896, NCT00300781, NCT01827267, NCT00266877	Marketed	Colorectal cancer, breast cancer, NSCLC	Iron modulation, system X_c_^−^ inhibition	Disrupts iron homeostasis, inhibits system X_c_^−^ → ↓ cystine → ↓ GSH → ↑ oxidative stress sensitises cells to ferroptosis
Lapatinib	NCT00536809, NCT00574171, NCT01184482, NCT04831528, NCT03418558	Marketed	Colorectal cancer, breast cancer, advanced solid tumours	Iron modulation, GPX4 downregulation	↑ intracellular iron and ROS, ↓ GPX4 expression → ↑ lipid peroxidation → ferroptosis

Classification of the listed drugs is based on reported modulation of ferroptosis-related pathways. Several compounds exert their primary antitumour effects through other established mechanisms and influence ferroptosis indirectly or in a context-dependent manner. Abbreviations: AIDS: acquired immunodeficiency syndrome, AML: acute myelogenous leukaemia, ATF4: activating transcription factor 4, Fe: iron, γ-GCS: gamma-glutamylcysteine synthetase, GPX4: glutathione peroxidase 4, GSH: glutathione, HCC: hepatocellular carcinoma, mtDNA: mitochondrial DNA, NRF2: nuclear factor erythroid 2-related factor 2, NSCLC: non-small cell lung cancer, PGK1: phosphoglycerate kinase 1, ROS: reactive oxygen species, SOD1: superoxide dismutase 1. Arrows indicate relative changes or directional effects (↑ increase, ↓ decrease, → causative or regulatory relationship).

**Table 3:** Representative clinical trials of iron-chelating drugs in cancer.

Therapeutic Agent	Indicative Clinical Trials	Clinical Phase/Approval Status for Cancer	Tested Cancer Type	Relation to Ferroptosis	Mechanism Related to Ferroptosis
Iron-Chelating Agents					
Deferoxamine	NCT05300958, NCT05184816	I, II	Metastatic TNBC, leptomeningeal metastases from solid tumours	Iron chelation	Binds free iron → altered iron homeostasis and lipid peroxidation for ferroptosis
Deferasirox	NCT02413021, NCT02341495, UMIN000013451	I, II	AML, ALL, HCC	Iron chelation	Binds free iron → ↓ iron availability for lipid peroxidation in ferroptosis
Deferiprone	NCT02477631, Other clinical trial examples in [245,246]	Pilot, ΙΙ	Myelodysplastic syndrome	Iron chelation	Binds free iron → ↓ iron availability for lipid peroxidation in ferroptosis
N-acetylcysteine	NCT04982146, NCT05123365, clinical trial in [265]	Pilot, I, II	Breast cancer, pseudomyxoma peritonei, myeloproliferative neoplasms	Iron chelation	Binds free iron → ↓ iron availability for lipid peroxidation in ferroptosis, increases intracellular GSH

Abbreviations: ALL: acute lymphoblastic leukaemia, AML: acute myelogenous leukaemia, HCC: hepatocellular carcinoma, GSH: glutathione, TNBC: triple-negative breast cancer. Arrows indicate relative changes or directional effects (↓ decrease, → causative or regulatory relationship).

## Data Availability

No new data were created or analysed in this study.

## Abbreviations

The following abbreviations are used in this manuscript:

ACSL3	Acyl-CoA synthetase long-chain family member 3
ACSL4	Acyl-CoA synthetase long-chain family member 4
AIDS	Acquired immunodeficiency syndrome
AML	Acute myelogenous leukaemia
AP-1	Activator protein-1
ApoE	Apolipoprotein E
ATF3	Activating transcription factor 3
ATF4	Activating transcription factor 4
BRCA	Breast cancer susceptibility gene (BRCA1/BRCA2)
CAF	Cancer-associated fibroblast
CNNM4	Cyclin M4 (magnesium transporter)
CNSI-Fe	Carbon nanoparticle-loaded iron(II) system
CRC	Colorectal cancer
DF	Deferoxamine
DFRA	Deferasirox
EGFR	Epidermal growth factor receptor
ELOVL6	Elongation of very long-chain fatty acids protein 6
EMT	Epithelial–mesenchymal transition
ERK1/2	Extracellular signal-regulated kinase 1/2
FABPs	Fatty acid-binding proteins
Fe	Iron
FSP1	Ferroptosis suppressor protein 1
FTH	Ferritin heavy chain
FTL	Ferritin light chain
GBM	Glioblastoma
γ-GCS	Gamma-glutamylcysteine synthetase
GPX4	Glutathione peroxidase 4
GSH	Glutathione
4-HNE	4-Hydroxynonenal
HCC	Hepatocellular carcinoma
HIF	Hypoxia-inducible factor
JNK	c-Jun N-terminal kinase
KRAS	Kirsten rat sarcoma viral oncogene homolog
L1	Deferiprone
LIP	Labile iron pool
LMWt Fe	Low-molecular-weight intracellular iron pool
LMWt PFe	Low-molecular-weight plasma iron pool
lncRNA	Long non-coding RNA
METTL3	Methyltransferase-like 3
MRI	Magnetic resonance imaging
mtDNA	Mitochondrial DNA
MUFA	Monounsaturated fatty acid
ncRNA	Non-coding RNA
NDRG1	N-MYC downstream-regulated gene 1
NK	Natural killer cells
NRF2	Nuclear factor erythroid 2-related factor 2
NSCLC	Non-small cell lung cancer
PARP	Poly(ADP-ribose) polymerase
PDAC	Pancreatic ductal adenocarcinoma
PGK1	Phosphoglycerate kinase 1
PUFA	Polyunsaturated fatty acid
RAS	Rat sarcoma viral oncogene family
RCC	Renal cell carcinoma
ROS	Reactive oxygen species
SIRT3	Sirtuin 3
SLC40A1	Solute carrier family 40 member 1 (ferroportin)
SLC7A11	Solute carrier family 7 member 11
SNHG3	Small nucleolar RNA host gene 3
SOD1	Superoxide dismutase 1
SREBP2	Sterol regulatory element-binding protein 2
STAT3	Signal transducer and activator of transcription 3
STEAP4	Six-transmembrane epithelial antigen of prostate 4
STING	Stimulator of interferon genes
TAM	Tumour-associated macrophage
TAN	Tumour-associated neutrophil
TAZ	Transcriptional coactivator with PDZ-binding motif
TME	Tumour microenvironment
TNBC	Triple-negative breast cancer
Treg	Regulatory T cell
TrR	Transferrin receptor 1
YAP	Yes-associated protein
